# Long sleep duration, cognitive performance, and the moderating role of depression: A cross‐sectional analysis in the Framingham Heart Study

**DOI:** 10.1002/alz.70160

**Published:** 2025-04-21

**Authors:** Vanessa M. Young, Rebecca Bernal, Andree‐Ann Baril, Joy Zeynoun, Crystal Wiedner, Carlos Gaona, Alexa Beiser, Antonio L. Teixeira, Arash Salardini, Matthew P. Pase, Jayandra Jung Himali, Sudha Seshadri

**Affiliations:** ^1^ Glenn Biggs Institute for Alzheimer's & Neurodegenerative Diseases, University of Texas Health Science Center at San Antonio San Antonio Texas USA; ^2^ Graduate School of Biomedical Sciences University of Texas Health Science Center at San Antonio San Antonio Texas USA; ^3^ School of Social and Behavioral Sciences Arizona State University Phoenix Arizona USA; ^4^ The Framingham Heart Study Framingham Massachusetts USA; ^5^ Research Center of the CIUSSS‐NIM, Hôpital du Sacré‐Coeur de Montréal Montreal Quebec Canada; ^6^ Department of Medicine University of Montreal Montreal Quebec Canada; ^7^ Department of Neurology Boston University Chobanian & Avedisian School of Medicine Boston Massachusetts USA; ^8^ Department of Biostatistics Boston University School of Public Health Boston Massachusetts USA; ^9^ Department of Neurology University of Texas Health Science Center at San Antonio San Antonio Texas USA; ^10^ Turner Institute for Brain and Mental Health Monash University Clayton Victoria Australia; ^11^ Department of Population Health Sciences University of Texas Health Science Center San Antonio Texas USA

**Keywords:** cognitive function, depression, sleep duration

## Abstract

**INTRODUCTION:**

We investigated whether depression modified the associations between sleep duration and cognitive performance.

**METHODS:**

We examined the associations between sleep duration and cognition in 1853 dementia‐and‐stroke‐free participants (mean age 49.8 years, [range 27–85]; 42.7% male). Participants were categorized into four groups: no depressive symptoms, no antidepressants; depressive symptoms without antidepressant use; antidepressant use without depressive symptoms; and depressive symptoms and antidepressant use.

**RESULTS:**

Long sleep was associated with reduced overall cognitive function (β ± standard error = −0.25 ± 0.07, *p* < 0.001), with strongest effects in those with depressive symptoms using (−0.74 ± 0.30, *p* = 0.017) and not using antidepressants (−0.60 ± 0.26, *p* = 0.024). Weaker but significant effects were observed in those without depressive symptoms (−0.18 ± 0.09, *p* = 0.044). No significant associations were observed in participants using antidepressants without depressive symptoms.

**DISCUSSION:**

Associations between sleep duration and cognitive performance are strongest in individuals with depressive symptoms, regardless of antidepressant use. Future research should elucidate underlying mechanisms and temporal relationships.

**Highlights:**

- Sleeping ≥ 9 hours/night was associated with worse cognitive performance.
- This association was stronger among those with depression.
- Long sleepers were more likely to report symptoms of depression.
- Sleep may be a modifiable risk for cognitive decline in people with depression.

## 1 BACKGROUND

There is an increasing recognition of the significance of sleep as a vital physiological process for brain health.^1^, ^2^ Disturbances in sleep duration and patterns are observed across the lifespan and are present in both normal and pathological aging.^3^, ^4^, ^5^ These disturbances contribute to an increased risk of cognitive deficits and Alzheimer's disease (AD).^6^ The Global Council on Brain Health recommends 7 to 8 hours of nightly sleep for adults to preserve brain health.^7^ Several studies have suggested that both excessive and insufficient sleep relative to the prescribed duration are linked to impairments in cognitive domains, including memory, attention, and executive functioning.^8^, ^9^, ^10^, ^11^, ^12^ However, evidence remains inconsistent across the literature with prior research predominantly focused on midlife and older adulthood. For example, a large pooled cohort study of > 28,000 people in mid to late life found that sleep durations of < 4 or > 10 hours were associated with faster cognitive decline, suggesting an inverted U‐curve relationship between sleep duration and cognition.^13^ In contrast, a cross‐sectional study of healthy middle‐aged subjects and a longitudinal study of older adults reported an association solely between short sleep duration (< 6 hours/night) and diminished cognition.^14^, ^15^, ^16^ Other cross‐sectional and longitudinal studies have only found prolonged sleep (≥ 9 hours/night) correlated with cognitive deterioration.^17^, ^18^ These inconsistencies suggest that the relationship between sleep duration and cognition may vary across the adult lifespan due to factors such as age and other health differences, such as depression.

Depression, a modifiable risk factor for cognitive decline,^19^ often co‐occurs with sleep disorders.^20^ The association between sleep disorders and depression is well established, with ≈ 90% of people with depression reporting problems with sleep.^21^ Sleep disorders are believed to precede depression rather than the contrary,^21^, ^22^ with insomnia being the most frequent sleep disorder associated with depressive symptoms.^23^ Depression‐related alterations in sleep architecture, such as reduced slow‐wave sleep (SWS) and rapid eye movement (REM) sleep,^24^ have been linked to cognitive impairment.^25^ The cognitive effects of depression appear to persist even after remission of depressive symptoms,^26^ contributing to a heightened vulnerability to dementia later in life.^27^ This complex relationship suggests that depression may modify the relationship between sleep and cognition, potentially amplifying the cognitive impact of inadequate sleep duration.

Antidepressant treatment adds further complexity to these relationships due to its distinct and sometimes opposing effects.^28^, ^29^ For instance, selective serotonin reuptake inhibitors (SSRIs) are associated with treatment‐emergent difficulty initiating or maintaining sleep^30^ while antidepressants with sedating properties like doxepin and trazodone are linked to excessive sleepiness.^30^ These medication‐induced changes in sleep duration can have varying effects on cognitive performance. Some antidepressants may have positive effects on cognitive function, with vortioxetine showing significant improvements in psychomotor speed and duloxetine showing benefits for delayed recall.^31^ In contrast, tricyclic antidepressants can potentially contribute to adverse side effects, such as worse delayed recall and psychomotor speed.^31^

The high prevalence of sleep disturbances and cognitive deficits in depression, along with varying effects of antidepressants and persistence of cognitive impairment after remission, suggests a complex interplay among these factors. While past research examined depression as a mediator focusing on mid‐life to later adulthood,^32^ this study investigates effect modification to capture changes in the strength of association between sleep duration and cognition across four subgroups: (1) no depressive symptoms/antidepressant use, (2) antidepressant use without current depressive symptoms, (3) no reported antidepressant use with depressive symptoms, and (4) antidepressant use with depressive symptoms. This novel stratified approach enables the detection of clinically relevant differences in sleep–cognition associations that could guide treatment approaches for managing cognitive function in those with depression.

We examined cross‐sectional associations between self‐reported nighttime sleep duration (short, average, and long) and cognitive performance in dementia‐ and stroke‐free adults enrolled in the Framingham Heart Study (FHS).^33^ We further explored whether depressive symptoms modified these associations. Our sample included adults, with participant ages ranging from 27 to 85 years, affording the opportunity to examine sleep–cognition associations and the effect of depressive symptoms and antidepressant use across a broader age spectrum. We hypothesized that (1) both long and short self‐reported nighttime sleep durations would be associated with poorer cognitive performance, and (2) that the relationship between sleep duration and cognitive performance would be stronger in individuals with depressive symptoms regardless of antidepressant usage, relative to those without depressive symptoms.

## 2 METHODS

### 2.1 Sample

**FIGURE 1 alz70160-fig-0001:**
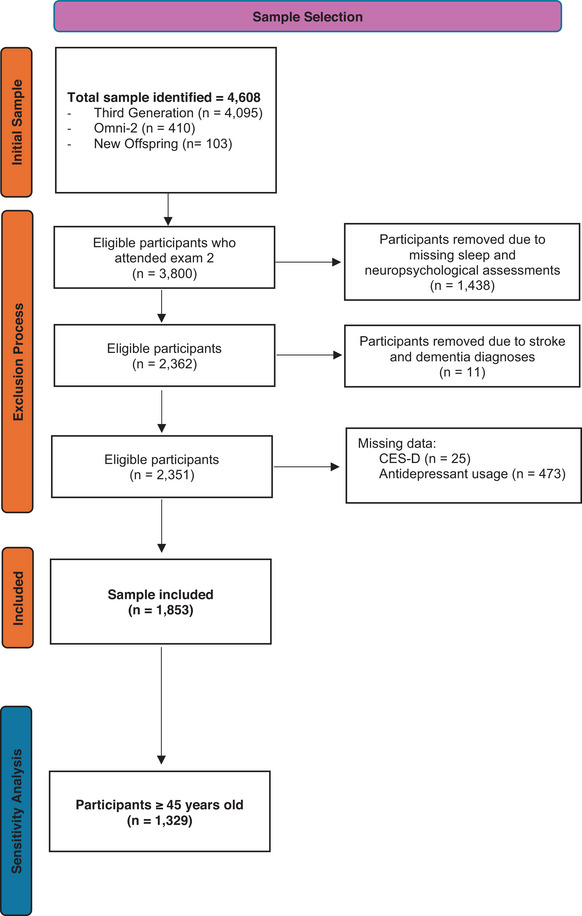
Flowchart of the sample selection process. The flowchart illustrates the sample selection from the initial population to the final analysis cohort. Each exclusion step accounts for missed visits, incomplete assessments, stroke or dementia, and missing depression data, resulting in a final cohort of 1853 participants. A sensitivity analysis was conducted on 1329 participants aged ≥ 45 years. CES‐D, Center for Epidemiologic Studies Depression Scale.

The FHS is a multi‐generational, longitudinal study started in 1948 with clinical examinations completed approximately every 4 years. We included 1853 participants from the FHS Third Generation, Omni 2, and New Offspring Spouse cohorts. The study included dementia‐ and stroke‐free participants who completed their second clinical examination cycle between 2008 and 2011 and agreed to undergo neuropsychological testing as part of the FHS ancillary study. We excluded participants with dementia or stroke, as extensive neurodegeneration in these conditions could have been confounding.^34^ Figure 1 illustrates the sample selection. This study was conducted in accordance with the Declaration of Helsinki. The institutional review board at Boston Medical Center approved the study protocol. All participants signed the informed consent.

### 2.2 Sleep assessment

Habitual self‐reported sleep duration was assessed during in‐clinic visits. The participants responded to a prompt that asked them to list the number of hours they typically sleep. We classified sleep duration into three categories: short sleep duration (< 6 hours), average sleep duration (between 6 and 9 hours) for the reference group, and long sleep duration (> 9 hours).^35^

**RESEARCH IN CONTEXT**

1. **Systematic review**: We conducted a systematic search on PubMed and Google Scholar for peer‐reviewed articles using keyword combinations related to sleep, cognition, and depression. Existing evidence reveals mixed findings on the relationship between sleep duration and cognition, with limited studies examining the role of depression on this association.
2. **Interpretation**: We observed that only long sleep duration (≥ 9 hours) was associated with poorer global cognition, executive function, visuospatial memory, and verbal learning/memory. Depression moderated this association, showing stronger negative effects of long sleep on cognition in individuals with depressive symptoms, regardless of antidepressant use. This suggests that long sleep duration may serve as an early indicator or risk factor for cognitive decline in those with depressive symptoms.
3. **Future directions**: Longitudinal studies with objective and subjective sleep assessments across diverse populations are needed to clarify how depression and its treatment influence the relationship between sleep duration and cognitive decline and support clinical strategies.

### 2.3 Cognitive assessments

Trained neuropsychological raters assessed cognitive function using a comprehensive and standardized test battery ≈ 1.7 ± 1.0 years after their clinical examination.^36^ In this study, we included the following tests: Trail Making Test Parts A and B,^37^ Visual Reproduction and Logical Memory Tests (sum of immediate and delayed recall scores) from the Weschler Memory Scale—Revised,^38^ and the Similarities Test from the Wechsler Adult Intelligence Scale.^39^ The tests assessed attention, processing speed, and executive function; visuospatial memory; verbal learning and memory; and verbal reasoning, respectively. Trail Making Test Parts A and B were log‐transformed and reversed so that a higher score indicated better performance for all cognitive tests. A global cognitive score was derived from a principal component analysis,^40^ combining weighted loadings of the Similarities Test, Visual Reproduction, Logical Memory Test, and Trail Making Test Part B.[Fig alz70160-fig-0001]

### 2.4 Measures of depression

We used the 20‐item Center for Epidemiologic Studies Depression (CES‐D) Scale^41^ to assess depressive symptoms. We defined depression as either a score of 16 on the CES‐D or the use of antidepressant medications (anatomical therapeutic chemical [ATC] code N06A).^42^ We then categorized the participants into four groups: (1) controls: those not using antidepressants and without depressive symptoms (CES‐D < 16), (2) those using antidepressants and without depressive symptoms (CES‐D < 16),; (3) those not using antidepressants with depressive symptoms (CES‐D ≥ 16), and (4) those using antidepressants and with depressive symptoms (CES‐D ≥ 16). The four‐category classification affords clinical nuances in the relationship between depressive symptoms, treatment status, and cognitive outcomes that may be masked when using a binary classification (yes versus no) for overall prevalence estimates.

### 2.5 Covariates

The following covariates, potentially associated with sleep, depression, or cognitive function, were incorporated into two separate statistical models:

1. Age (years) and age squared (years^2^) at the time of neuropsychological testing.
2. Education: high school or less, some college, or college degree.
3. Sex: female or male.
4. Cohort: Third Generation, Omni 2, and New Offspring Spouse cohorts. Analyses were adjusted for cohort effects to account for ethnic composition and generation differences across recruitment periods.^43^
5. Time interval between sleep duration questionnaires and neuropsychological testing.
6. Hypertension characterized according to the Seventh Report of the Joint National Committee on Prevention, Detection, Evaluation, and Treatment of High Blood Pressure, as stage I hypertension or worse, defined by a systolic blood pressure (SBP) of ≥ 140 mmHg, a diastolic blood pressure (DBP) of ≥ 90 mmHg, or the use of antihypertensive medication.^44^
7. Ethylenediaminetetraacetic acid plasma aliquots were analyzed daily at the FHS laboratory to measure: (a) total cholesterol level (mg/dL), (b) high‐density lipoprotein (HDL) level (mg/dL),^45^ and (c) triglycerides (mg/dL).
8. C‐reactive protein (CRP) levels were categorized as low < 1 mg/L, average 1 to 3 mg/L, and high > 3 mg/L, according to the American Heart Association guidelines.^46^
9. Body mass index (BMI) calculated using weight in kilograms and divided by the square of height in meters (m^2^).
10. Apolipoprotein E (*APOE*) ɛ4 allele carrier status: those with at least one ɛ4 allele (as determined by polymerase chain reaction) versus those with none.

### 2.6 Statistical analyses

We used SAS V.9.4 (SAS Institute) to perform statistical analyses and considered statistical significance at *p* < 0.05, except for the test of interactions for which *p* < 0.10 was used to account for their inherently lower statistical power compared to main effects as done in previous work.^47^ We treated self‐reported sleep duration (short, average, and long sleep duration) and depression (control, antidepressant usage/CES‐D < 16, no antidepressant usage/CES‐D ≥ 16, and antidepressant usage CES‐D ≥ 16), as categorical variables. Group differences were assessed using analysis of variance for continuous variables and chi‐square tests for categorical variables.

First, we used multivariable linear regression models to examine the associations between the three sleep categories and global cognition. Secondary analyses assessed the associations between sleep duration categories and individual cognitive tasks. We used two sequential models to examine these associations. Model 1 was adjusted for basic demographic and study design confounders: age and age^2^ at neuropsychological testing, education, sex, time between sleep duration questionnaires and neuropsychological testing, and cohort. In Model 2, we additionally included cardiovascular and metabolic factors that may mediate the relationship between sleep and cognition:^48^, ^49^, ^50^ hypertension, total cholesterol to HDL ratio, triglycerides, CRP level, BMI, and *APOE* ɛ4 status.

We explored effect modification by depression group for the associations between sleep duration and cognitive performance by adding an interaction term with sleep in Model 1. When a statistically significant interaction was observed, we stratified the association between sleep duration and cognitive performance by depression group.

### 2.7 Sensitivity analysis

In the primary models between sleep duration and cognitive performance, we conducted a sensitivity analysis limiting the sample to middle‐aged and older adults (cut‐off ≥ 45 years, following the age groups defined by the National Library of Medicine^51^) to examine whether the sleep duration–cognition association and potential effect modification by depression group remained consistent when excluding younger adults from the analysis.

## 3 RESULTS

### 3.1 Sample characteristics

The sample consisted of 1853 adults with an average age of 49.8 years (age range 27–85). The sample was 42.7% male, 95.3% White, and 57.2% college educated. Most of the participants reported average sleep durations of 6 to 9 hours (68.9%), followed by short sleep (≤ 6 hours; 24.3%) and long sleep (≥ 9 hours; 6.8%). Depression, defined as CES‐D ≥ 16 or the use of antidepressant medications, was present in 448 (24.2%) participants: 315 (17.0%) reported using antidepressants, while 198 (11.0) had a CES‐D score of ≥ 16. The group with long sleep duration tended to have a higher proportion of depression (*n* = 55, 44.0%) compared to the short (*n* = 106, 23.5%) and average (*n* = 287, 22.5%) sleep groups. Table 1 presents the overall sample's characteristics and between sleep group differences, and Table 2 shows the performance on domain‐specific cognitive tests categorized by sleep duration.

**TABLE 1:** Sample characteristics.

		Short sleep	Average sleep	Long sleep	
	Whole sample	(≤ 6 hours)	(> 6– < 9 hours)	(≥ 9 hours)	*p* ^*^
*N*, *n* (%)	1853	451 (24.3)	1277 (68.9)	125 (6.8)	
Age at NP, years	49.84 (9.2)	50.57 (8.2)	49.57 (9.3)	49.92 (11.1)	0.137
Male, *n* (%)	791 (42.7)	240 (53.2)	511 (40.0)	40 (32.0)	**<0.001**
Ancestry/ethnicity, *n* (%)					**0.019**
White	1760 (95.3)	422 (93.6)	1218 (95.8)	121 (96.8)	
Black	19 (1.0)	12 (2.7)	7 (0.6)	0	
Hispanic	33 (1.8)	7 (1.6)	22 (1.7)	4 (3.2)	
Other	35 (1.9)	10 (2.2)	24 (1.9)	1 (0.8)	
Education, n (%)					**0.002**
Up to high school	262 (14.1)	81 (18.0)	162 (12.7)	19 (15.2)	
Some college	532 (28.7)	153 (33.9)	338 (26.5)	41 (32.8)	
College degree	1059 (57.2)	217 (48.1)	777 (60.9)	65 (52.0)	
Time interval^a^, years	1.69 (1.0)	1.61 (0.9)	1.73 (1.0)	1.59 (1.0)	0.064
*APOE* ε4 carriers, *n* (%)	384 (21.8)	89 (20.7)	273 (22.5)	22 (18.8)	0.883
Total cholesterol, mg/dL	185.98 (35.0)	185.28 (35.0)	186.05 (35.2)	187.71 (33.7)	0.782
HDL, mg/dL	60.53 (17.9)	58.26 (17.9)	61.01 (17.7)	63.67 (19.7)	**0.003**
Systolic BP, mm/Hg	116 (14.1)	117 (13.6)	116 (14.1)	116 (16.9)	0.204
HTN treatment, *n* (%)	390 (21.1)	101 (22.5)	259 (20.4)	30 (24.2)	0.786
Stage 1 hypertension, *n* (%)	473 (25.7)	121 (27.0)	318 (25.0)	34 (27.4)	0.695
Triglycerides, mg/dL	112.8 (85.8)	116.89 (95.2)	111.03 (83.3)	116.16 (75.0)	0.416
CRP, mg/dL	2.83 (4.5)	2.72 (3.9)	2.73 (4.4)	4.29 (7.2)	**0.001**
BMI	28.19 (5.8)	29.24 (5.7)	27.83 (5.7)	28.15 (6.4)	**<0.001**
Depression status, *n* (%)	448 (24.2)	106 (23.5)	287 (22.5)	55 (44.0)	**0.004**
Antidepressant usage	315 (17.0)	57 (12.6)	215 (16.8)	43 (34.4)	**<0.001**
Depression, CES‐D ≤ 16	198 (10.7)	65 (14.4)	110 (8.6)	23 (18.4)	**0.309**
Depression groups					**0.122**
No antidepressant usage/CES‐D < 16^b^	1405 (75.8)	345 (76.5)	990 (77.5)	70 (56.0)	
Antidepressant usage/CES‐D < 16^c^	250 (13.5)	41 (9.1)	177 (13.9)	32 (25.6)	
No antidepressant usage/CES‐D ≥ 16^d^	133 (7.2)	49 (10.9)	72 (5.6)	12 (9.6)	
Antidepressant usage/CES‐D ≥ 16^e^	65 (3.5)	16 (3.6)	38 (3.0)	11 (8.8)	

*Note*: All values represent mean (standard deviation) unless otherwise indicated by n (%).

Bold values indicate statistical significance.

Abbreviations: *APOE*, apolipoprotein E; BMI, body mass index; BP, blood pressure; CRP, C‐reactive protein; HDL, high‐density lipoprotein cholesterol; HTN, hypertension; NP, neuropsychological assessment.

^a^ Time interval between self‐reported sleep duration and cognitive tests.

^b^ No antidepressant usage and no depressive symptoms (CES‐D < 16).

^c^ Antidepressant usage without depressive symptoms (CES‐D < 16).

^d^ No antidepressant usage with depressive symptoms (CES‐D ≥ 16).

^e^ Antidepressant usage and with depressive symptoms (CES‐D ≥ 16).

* Group differences were analyzed using analysis of variance for continuous variables and chi‐square tests for categorical variables.

**TABLE 2:** Sample cognitive characteristics.

	Whole sample	Short sleep	Average sleep	Long sleep	*p*
		(≤ 6 hours)	(> 6– < 9 hours)	(≥ 9 hours)	
*N*, *n* (%)	1853	451 (24.3)	1277 (68.9)	125 (6.8)	
Global cognition,^a^ median (Q1, Q3)	0.45 (−0.12,0.98)	0.32 (−0.19,0.91)	0.49 (−0.06,1.01)	0.15 (−0.49,0.88)	**<0.001**
Trails Part A, min, median (Q1, Q3)	0.40 (0.33,0.48)	0.40 (0.33,0.48)	0.40 (0.33,0.48)	0.40 (0.32,0.55)	0.189
Trails Part B, min, median (Q1, Q3)	0.97 (0.77,1.22)	1.00 (0.77,1.30)	0.95 (0.75,1.20)	0.97 (0.80,1.33)	0.**001**
Visual reproduction^b^, *n* correct	17.99 (4.89)	17.56 (5.09)	18.31 (4.70)	16.22 (5.56)	**<0.001**
Logical memory^b^, *n* correct	24.46 (6.74)	24.14 (6.59)	24.71 (6.74)	23.12 (7.12)	0.**022**
Similarities test, *n* correct	17.20 (3.15)	17.08 (3.14)	17.27 (3.17)	16.95 (3.01)	0.373

Bold values indicate statistical significance.

Abbreviations: Min, minutes; Trails, Trail Making Test.

^a^ Weighted score units.

^b^ Sum of immediate and delayed recall scores. All values represent mean (SD) unless otherwise indicated by median (Q1, Q3).

When examining sleep duration across the four depression groups (see Table 3), those using antidepressants and with depressive symptoms (CES‐D ≥ 16) reported the highest proportion of long sleep (*n* = 11; 16.9%), followed by those using antidepressants but without depressive symptoms (*n* = 32; 12.8%). In contrast, the control group (no antidepressant usage and without depressive symptoms) had the lowest proportion of long sleepers (*n* = 70; 5.0%). See Table 4 for performance on domain‐specific cognitive tests by depression group.

**TABLE 3:** Sample characteristics by depression categories.

	No antidepressant usage/CES‐D < 16^a^	Antidepressant usage/CES‐D < 16^b^	No antidepressant usage/CES‐D ≥ 16^c^	Antidepressant usage/ CES‐D ≥16^d^	*p*
*N*, *n* (%)	1405 (75.8)	250 (13.5)	133 (7.2)	65 (3.5)	
Age at NP, years	49.87 (9.3)	50.38 (8.7)	48.51 (8.8)	49.74 (8.8)	0.299
Male, *n* (%)	656 (46.7)	59 (23.6)	54 (40.6)	22 (33.8)	**<0.001**
Ancestry/ethnicity, *n* (%)					0.371
White	1330 (95.0)	243 (97.2)	124 (93.2)	63 (98.4)	
Black	14 (1.0)	2 (0.0)	3 (2.3)	0	
Hispanic	26 (1.9)	3 (1.2)	3 (2.3)	1 (1.6)	
Other	30 (2.1)	2 (0.8)	3 (2.3)	0	
Education, *n* (%)					**<0.001**
Up to high school	179 (12.7)	38 (15.2)	28 (21.1)	17 (26.2)	
Some college	390 (27.8)	78 (31.2)	40 (30.1)	24 (36.9)	
College degree	836 (59.5)	134 (53.6)	65 (48.9)	24 (36.9)	
Time interval^e^, years	1.74 (1.0)	1.57 (0.9)	1.47 (0.9)	1.59 (1.1)	**0.003**
*APOE* ɛ4 carriers, *n* (%)	293 (21.9)	54 (22.6)	24 (18.9)	13 (21.7)	0.678
Total cholesterol, mg/dL	184.91 (35.0)	191.38 (33.4)	185.77 (36.6)	188.69 (32.4)	0.055
HDL, mg/dL	60.14 (17.9)	63.06 (18.1)	60.25 (18.6)	59.69 (15.8)	0.123
Systolic BP, mm/Hg	116 (14.1)	115.8 (13.3)	115 (15.0)	117 (15.2)	0.742
HTN treatment, *n* (%)	287 (20.5)	54 (21.7)	33 (24.8)	16 (24.6)	0.184
Stage 1 hypertension, *n* (%)	347 (24.8)	62 (24.9)	43 (32.6)	21 (32.3)	**0.042**
Triglycerides, mg/dL	111.5 (84.3)	113.7 (64.6)	110.6 (70.7)	141.6 (171.7)	0.051
CRP, mg/dL	2.72 (4.6)	4.28 (5.3)	2.79 (3.6)	4.28 (5.3)	**0.043**
BMI	27.89 (5.4)	28.61 (6.2)	29.80 (7.7)	29.91 (7.1)	**<0.001**
Sleep categories					0.122
Short sleep (≤ 6 hours)	345 (24.6)	41 (16.4)	49 (36.8)	16 (24.6)	
Average sleep (> 6– < 9 hours)	990 (70.5)	177 (70.8)	72 (54.1)	38 (58.5)	
Long sleep (≥ 9 hours)	70 (5.0)	32 (12.8)	12 (9.0)	11 (16.9)	

*Note*: All values represent mean (standard deviation), unless otherwise indicated by *n* (%).

Bold values indicate statistical significance.

Abbreviations: *APOE*, apolipoprotein E; BMI, body mass index; BP, blood pressure; CES‐D, Center for Epidemiologic Studies Depression Scale; CRP, C‐reactive protein; HDL, high‐density lipoprotein cholesterol; HTN, hypertension; NP, neuropsychological assessment.

^a^ No antidepressant usage and no depressive symptoms (CES‐D < 16).

^b^ Antidepressant usage without depressive symptoms (CES‐D < 16).

^c^ No antidepressant usage with depressive symptoms (CES‐D ≥ 16).

^d^ Antidepressant usage and with depressive symptoms (CES‐D ≥ 16).

^e^ Time interval between self‐reported sleep duration and cognitive tests.

**TABLE 4:** Cognitive characteristics by depression categories.

	No antidepressant usage/CES‐D < 16^a^	Antidepressant usage/CES‐D < 16^b^	No antidepressant usage/CES‐D ≥ 16^c^	Antidepressant usage/CES‐D ≥16 ^d^	*p*
*N*, *n* (%)	1405 (75.82)	250 (13.49)	133 (7.18)	65 (3.51)	
Global cognition^e^, median (Q1, Q3)	0.47 (−0.09, 0.99)	0.41 (−0.19, 0.91)	0.42 (−0.33, 0.96)	0.12 (−0.38, 0.75)	**0.002**
Trails Part A, min, median (Q1, Q3)	0.40 (0.33, 0.48)	0.40 (0.33, 0.48)	0.42 (0.33, 0.50)	0.42 (0.32, 0.52)	0.263
Trails Part B, min, median (Q1, Q3)	0.95 (0.75, 1.22)	0.98 (0.78, 1.20)	0.95 (0.75, 1.27)	1.12 (0.88, 1.45)	**<0.001**
Visual reproduction^f^, *n* correct	18.21 (4.81)	17.44 (4.64)	17.70 (5.53)	15.88 (5.56)	**<0.001**
Logical memory^f^, *n* correct	24.44 (6.70)	24.99 (7.03)	24.17 (6.53)	23.46 (7.04)	0.361
Similarities test, *n* correct	17.26 (3.10)	17.12 (2.97)	16.87 (3.42)	16.94 (4.10)	0.471

*Note*: All values represent mean (standard deviation) unless otherwise indicated by median (Q1, Q3).

Bold values indicate statistical significance.

Abbreviations: CES‐D, Center for Epidemiologic Studies Depression Scale; Min, minutes; Trails, Trail Making Test.

^a^ No antidepressant usage and no depressive symptoms (CES‐D < 16).

^b^ Antidepressant usage without depressive symptoms (CES‐D < 16).

^c^ No antidepressant usage with depressive symptoms (CES‐D ≥ 16).

^d^ Antidepressant usage and with depressive symptoms (CES‐D ≥ 16).

^e^ Weighted score units.

^f^ Sum of immediate and delayed recall scores combined.

### 3.2 Association between sleep and cognition

#### 3.2.1 Long sleep duration was associated with global cognition

Table 5 presents the association between short and long sleep durations and global cognitive performance, compared to average sleep duration (> 6 or < 9 hours vs. 6–9 hours). Long sleep duration (≥ 9 hours) had a statistically significant association with poorer global cognition (*β ± *standard error* =* −0.25 ± 0.07, *p* < 0.001), as measured by the global cognitive score, relative to those with average sleep duration. The association between short sleep duration (≤ 6 hours) and global cognition did not reach statistical significance (−0.03 ± 0.04, *P *= 0.465). In Model 2, the pattern of associations between long sleep duration and global cognition remained consistent with Model 1.

**TABLE 5:** Association between short and long sleep duration and cognitive scores.

		Sleep duration categories				
		Average	Short sleep		Long sleep	
		(> 6– < 9 hours)	(≤ 6 hours)		(≥ 9 hours)	
Cognition	Model		*β* ± SE	*p*	*β* ± SE	*p*
Global cognition	1	REF	−0.03 ± 0.04	0.465	−**0.25 ± 0.07**	**<0.001**
	2	REF	−0.04 ± 0.04	0.280	−**0.27 ± 0.07**	**<0.001**
Trails Part A	1	REF	−0.001 ± 0.02	0.959	−0.03 ± 0.03	0.205
	2	REF	−0.01 ± 0.02	0.550	−0.03 ± 0.03	0.279
Trails Part B	1	REF	−0.02 ± 0.02	0.235	−**0.09 ± 0.03**	**0.009**
	2	REF	−0.04 ± 0.02	0.067	−**0.07 ± 0.03**	**0.041**
Visual reproduction	1	REF	−0.41 ± 0.25	0.104	−**1.80 ± 0.42**	**<0.001**
	2	REF	−0.45 ± 0.26	0.081	−**1.76 ± 0.44**	**<0.001**
Logical memory	1	REF	0.25 ± 0.35	0.480	−**1.50 ± 0.60**	**0.013**
	2	REF	0.22 ± 0.36	0.538	−**2.16 ± 0.62**	**<0.001**
Similarities test	1	REF	0.01 ± 0.17	0.954	−0.06 ± 0.28	0.839
	2	REF	0.002 ± 0.17	0.990	−0.11 ± 0.29	0.701

*Note*: Model 1 was adjusted for age and age squared at neuropsychological testing, education, sex, time between sleep duration questionnaires and neuropsychological testing, and cohort. Model 2 was further adjusted for hypertension, total cholesterol level to HDL ratio, triglycerides, C reactive protein level, body mass index, and *APOE* ɛ4 status.

Bold values indicate statistical significance.

Abbreviations: *APOE*, apolipoprotein E; HDL, high‐density lipoprotein; SE, standard error; Trails, Trail Making Test.

#### 3.2.2 Long sleep duration was associated with task‐specific cognitive domains

Long sleep duration (≥ 9 hours) was associated with significantly worse performance on Trail Making Test Part B (−0.09 ± 0.03, *P *= 0.009), Visual Reproduction (−1.80 ± 0.42, *p* < 0.001), and Logical Memory (−1.50 ± 0.60, *P *= 0.013) compared to the average sleep duration group. Long sleep was not associated with Trail Making Test Part A or the Similarities Test. Short sleep was not associated with any cognitive tasks. The results remained consistent in the fully adjusted model (see Table 5).

#### 3.2.3 Sleep duration interacts with depression in its association with cognition

We tested statistical interactions to determine whether depression groups modified the relationships between sleep duration categories and cognitive performance. We observed significant interactions indicating effects on global cognitive score (*p* = 0.028), Trail Making Test Part B (*p* = 0.012), Visual Reproduction (P*p* = 0.064), and Similarities Test (*p* = 0.027).

As shown in Table 6, in the control group (no antidepressants, CES‐D < 16), long sleep was associated with poorer global cognition (−0.18 ± 0.09, *p* = 0.044) and visual reproduction (−1.30 ± 0.55, *p* = 0.019), while short sleep showed no significant associations with any cognitive measure. In those with depressive symptoms (CES‐D ≥ 16) not using antidepressants, long sleep was associated with poorer performance across multiple domains: global cognition (−0.60 ± 0.26, *p* = 0.024), Trails Part B (−0.29 ± 0.13, *p* = 0.023), and visual reproduction (−3.70 ± 1.70, *P *= 0.031). Short sleep was not significantly associated with cognitive measures in this group. Among those using antidepressants with depressive symptoms (CES‐D ≥ 16), long sleep was associated with poorer global cognition (−0.74 ± 0.30, *p* = 0.017) and visual reproduction (−4.30 ± 1.89, *P *= 0.027). We observed no significant associations between short sleep and cognitive performance in individuals using antidepressants but with depressive symptoms. Finally, among participants using antidepressants but without depressive symptoms (CES‐D < 16), neither short nor long sleep duration was significantly associated with cognitive performance.

**TABLE 6:** Effect modification by depression group on the association between self‐reported sleep duration and cognitive scores.

		Global cognition		Trails Part B		Visual reproduction		Similarities	
	Sleep duration interaction	*p* = 0.028		*p* = 0.012		*p* = 0.064		*p* = 0.027	
		*β* ± SE	*p*	*β* ± SE	*p*	*β* ± SE	*p*	*β* ± SE	*p*
**No antidepressant usage/CES‐D < 16** ^a^	Average Sleep	REF		REF		REF		REF	
	Short sleep	−0.03 ± 0.04	0.522	−0.03 ± 0.02	0.235	−0.31 ± 0.28	0.270	−0.05 ± 0.19	0.776
	Long sleep	**−0.18 ± 0.09**	**0.044**	−0.04 ± 0.04	0.379	**−1.30 ± 0.55**	**0.019**	0.24 ± 0.37	0.512
**Antidepressant usage and CES‐D < 16** ^b^	Average sleep	REF		REF		REF		REF	
	Short sleep	−0.06 ± 0.13	0.632	−0.07 ± 0.06	0.273	−0.76 ± 0.74	0.304	−0.13 ± 0.50	0.790
	Long sleep	−0.07 ± 0.14	0.621	−0.03 ± 0.07	0.632	−0.46 ± 0.83	0.581	0.18 ± 0.55	0.748
**No antidepressant usage and CES‐D ≥ 16** ^c^	Average sleep	REF		REF		REF		REF	
	Short sleep	−0.12 ± 0.15	0.453	−0.05 ± 0.08	0.500	−1.23 ± 1.00	0.220	0.42 ± 0.60	0.490
	Long sleep	**−0.60 ± 0.26**	**0.024**	**−0.29 ± 0.13**	**0.023**	**−3.70 ± 1.70**	**0.031**	−1.15 ± 1.03	0.267
**Antidepressant usage and CES‐D ≥ 16** ^d^	Average sleep	REF		REF		REF		REF	
	Short sleep	0.23 ± 0.26	0.384	0.17 ± 0.12	0.164	0.04 ± 1.65	0.979	1.04 ± 1.17	0.375
	Long sleep	**−0.74 ± 0.30**	**0.017**	−0.25 ± 0.13	0.067	**−4.30 ± 1.89**	**0.027**	−1.95 ± 1.33	0.149

*Note*: Sleep categories: short ≤ 6 hours; average 7–8 hours; long ≥ 9 hours. Model 1 was adjusted for age and age^2^ at neuropsychological testing, education, sex, time between sleep duration questionnaires, and neuropsychological testing and cohort. No significant interaction was found with Trails Part A (*p* = 0.114), and Logical Memory (*p* = 0.624).

Abbreviation: CES‐D, Center for Epidemiologic Studies Depression Scale; SE, standard error; Trails, Trail Making Test.

^a^ No antidepressant usage and no depressive symptoms (CES‐D < 16).

^b^ Antidepressant usage without depressive symptoms (CES‐D < 16).

^c^ No antidepressant usage with depressive symptoms (CES‐D ≥ 16).

^d^ Antidepressant usage and with depressive symptoms (CES‐D ≥ 16).

#### 3.2.4 Sensitivity analysis

In a sensitivity analysis including middle‐aged and older adults (cut‐off ≥ 45 years; see Tables S1 and S2 in supporting information for sample characteristics and cognitive characteristics by age group), the pattern of associations between sleep duration and cognitive performance was largely consistent with the full sample results as shown in Table S3 in supporting information. Significant interactions indicating effect modification by the depression group were observed in the associations between sleep duration and global cognitive score (*p* = 0.009), Trails Part B (*p* = 0.038), Visual Reproduction (*p* = 0.050), and Similarities Test (*p* = 0.006). The most pronounced effects were seen in those using antidepressants and with depressive symptoms (CES‐D ≥ 16). Relative to average sleep duration, long sleep duration was associated with significantly worse performance in all four cognitive measures: global cognition (−1.10 ± 0.35, *p* = 0.004), Trails Part B (−0.36 ± 0.15, *p* = 0.023), Visual Reproduction (−6.59 ± 2.24, *p* = 0.005), and Similarities Test (−3.80 ± 1.69, *p* = 0.030). In contrast, for the control group, only Visual Reproduction showed a significant negative association with long sleep duration (−1.43 ± 0.68, *p* = 0.037). No significant associations were observed among those using antidepressants and no depressive symptoms (CES‐D < 16) and those not using antidepressants with depressive symptoms (CES‐D ≥16; see Table S4 in supporting information).

## 4 DISCUSSION

We investigated the association between self‐reported sleep duration and cognition, and whether depression modified this relationship. Relative to average sleep duration, only short sleep duration was associated with poorer performance in global cognition, executive function, visuospatial memory, and verbal learning/memory tasks. These findings suggest that sleeping ≥ 9 hours per night is associated with cognitive differences equivalent to 6.5 years of brain aging. Furthermore, the strongest negative associations were among individuals with depressive symptoms, regardless of antidepressant usage status (yes vs. no).

Our hypothesis posited that both long and short sleep durations correlate with poorer cognitive function, as current evidence points to a U‐shaped association between sleep duration and cognition.^8^, ^9^, ^10^, ^11^, ^12^ Our findings partially support this hypothesis, corroborating the association between long sleep duration, worse global cognition, and poorer performance in multiple cognitive domains.^8^, ^9^, ^10^, ^11^, ^12^ While trending in the expected direction, we did not find a statistically significant relationship between short sleep duration and cognitive performance. These results diverge from some studies that have identified short sleep as a risk factor for cognitive impairment,^8^, ^9^, ^10^, ^11^, ^12^ while aligning with others who reported a significant association between long sleep duration and poorer cognition only.^17^, ^18^ The precision of this association may increase with greater statistical power or in older populations in which short sleep and cognitive impairment may be more prevalent.

When exploring the role of depression in the sleep–cognition relationship, associations between long sleep and cognition were more pronounced in individuals with depressive symptoms (CES‐D ≥ 16) relative to those not using antidepressants and without depressive symptoms. Specifically, in those with no prescribed antidepressants but with depressive symptoms (CES‐D ≥ 16), long sleep was significantly associated with poorer performance in global cognition, executive function, and visuospatial memory. In those using antidepressants and with depressive symptoms (CES‐D ≥ 16), long sleep was associated with worse global cognitive scores and visuospatial memory. Conversely, no associations were found in those using antidepressants and potentially remitted depression (CES‐D < 16). While individuals in this group still report long sleep duration, antidepressant use may improve other critical dimensions of sleep health as described by Buysse's Ru‐SATED model (Regularity, Satisfaction, Alertness, Timing, Efficiency, and Duration), and as a result, cognitive outcomes.^52^ For instance, antidepressant use may reduce nighttime awakenings (efficiency), improve sleep satisfaction with restorative sleep, or stabilize sleep–wake timing.^28^ However, whether this observation reflects antidepressant treatment, symptom remission, or other unmeasured aspects of sleep health remains unclear, given the limitations of our sleep measures.

Our sensitivity analysis of middle‐aged and older adults (≥ 45 years) showed broader cognitive vulnerabilities among individuals using antidepressants and with depressive symptoms. Specifically, the associations between long sleep duration and cognitive performance extended beyond global cognition and visual reproduction to impairments in executive function and verbal abilities. This expanded pattern of cognitive deficits was unique to individuals with depressive symptoms using antidepressants. In contrast, among those without depressive symptoms and no antidepressant use the association with long sleep remained confined to visual reproduction. These age‐specific findings suggest two potential interpretations: (1) the combination of depression and long sleep duration may have increased negative effects on cognitive health in middle and later life, or (2) these patterns may represent prodromal manifestations of neurodegenerative diseases rather than independent risk factors. Despite excluding individuals with diagnosed dementia and stroke, subclinical neurodegenerative processes could be present in some participants, particularly in the upper age ranges. Longitudinal studies are warranted to elucidate whether these cognitive vulnerabilities represent cumulative effects of prolonged depressive symptoms and antidepressant use, early manifestations of neurodegenerative processes, or both. Such distinction would inform whether interventions should target sleep and mood symptoms directly or be integrated into comprehensive approaches for early detection and management of neurodegenerative disease.

These findings contrast with a recent study by Wang et al.,^32^ who found that long sleep duration was associated with better cognitive scores (Telephone Interview of Cognitive Status 10, word recall, figure drawing) in those with depressive symptoms (CES‐D_10_ ≥ 10) among 12,589 participants aged ≥ 45 from the China Health and Retirement Longitudinal Study (CHARLS). While both studies highlight the complex relationship among sleep, cognition, and depression, several factors could account for the divergent results. The cognitive assessments varied substantially, with our study using a comprehensive neuropsychological battery, potentially capturing subtle cognitive deficits associated with long sleep duration that could have been missed by the shorter screening measures used by Wang et al.^32^ In addition, differences in study populations, with Wang et al. concentrating on a Chinese cohort and our research predominantly involving White individuals residing in the United States, suggest possible environmental and cultural differences in sleep patterns and the manifestation of depression symptoms.^53^ Further research is required to shed light on these relationships across diverse populations.

The reciprocal association between sleep disturbances and depression may create a cycle in which disrupted sleep exacerbates depressed symptoms, which in turn influences sleep quality.^54^ Sleep disturbance may increase vulnerability to depression by altering neural sensitivity to inflammation^55^ and disrupting neurotransmitter systems involved in mood regulation, such as serotonin and dopamine.^56^ Conversely, depressive symptoms can disrupt sleep due to factors like worrying and difficulty coping with stress.^57^ Chronic stress and associated physiological changes, such as inflammation and dysregulation of the hypothalamic–pituitary–adrenal (HPA) axis, can further exacerbate depression and cognitive impairment.^58^, ^59^ In those with depression, the HPA axis is usually hyperactive, potentially leading to fragmented sleep and reduced SWS duration.^60^, ^61^ During SWS, the glymphatic system eliminates metabolic waste products such as amyloid beta and tau proteins.^62^, ^63^, ^64^ Fragmented sleep or reduced SWS duration^65^ can impede this clearance mechanism and potentially facilitate the buildup of neurotoxic proteins leading to cognitive deterioration. Future research using polysomnography and actigraphy can clarify the mechanisms that link long sleep duration to cognition.

Long sleep duration may be an early and potentially reversible biological marker of neurodegeneration and a risk factor for dementia onset later in life.^66^, ^67^ We observed that long sleep duration was associated with lower performance in specific cognitive domains, particularly executive function and visuospatial memory. These associations were present across our full age range (27–85 years) and may reflect broader relationships between sleep duration and cognition rather than neurodegenerative processes specifically. Longitudinal studies integrating multimodal biomarker assessments of sleep, neuropsychiatric symptoms, and AD pathology are needed to better understand these relationships and identify opportunities for therapeutic intervention.

Finally, significant associations between long sleep duration and poorer cognitive performance were found in those not using antidepressants and without depressive symptoms. This group had a much larger sample size compared to the other three depression groups, which increases statistical power and the likelihood of detecting even small effects. The effect sizes in this group, however, were smaller than in the groups with depressive symptoms. Hence, while the associations were statistically significant, they may not be clinically significant. Nonetheless, these associations highlight how the relationship between sleep duration and cognitive function is not exclusive to those with depressive symptoms. Underlying mechanisms operating independently of depression, such as sleep architecture, circadian rhythms,^68^ or subclinical depressive symptoms,^69^ may influence the link between sleep duration and cognition. A multidisciplinary care model involving sleep specialists, neuropsychologists, and mental health professionals may be valuable in enhancing early detection and intervention strategies to prevent accelerated cognitive deficits. Further research is needed to determine (1) which specific sleep interventions, such as cognitive behavioral therapy for insomnia, or light therapy, might be most effective and feasible to implement in clinical practice for people with depression and (2) whether existing interventions are effective or require adaptations to better address the unique sleep challenges faced by individuals with depression.

### 4.1 Strengths and limitations

Our comprehensive battery of neuropsychological tests enabled us to examine specific cognitive domains that may be preferentially associated with long sleep duration. Our categorization of depression, considering both symptom severity and antidepressant status, offered a more nuanced examination of how depressive symptoms and treatment might modify the sleep–cognition link. These findings highlight the need for targeted longitudinal and interventional research that incorporates objective sleep measures to further investigate sleep and depression as co‐occurrent modifiable risk factors for cognitive decline.

Our study has limitations. Its cross‐sectional design prevented us from determining the causality of the relationships between sleep and cognition. We used self‐reported measures to assess sleep duration, which can be subject to recall biases, including overestimation of actual time asleep versus time spent in bed. Some antidepressants can prolong sleep time and alter sleep patterns and architecture, potentially contributing to the poorer cognitive performance observed in individuals with long sleep duration and active depression.^31^ Finally, the sample primarily consisted of White participants, which limits the generalizability of the findings to more diverse populations. Longitudinal studies including diverse and ethnic backgrounds may enhance the applicability of findings to broader communities.

## 5 CONCLUSIONS

Long but not short sleep duration was associated with poorer global cognition and specific cognitive abilities like memory, visuospatial skills, and executive functions. These associations were stronger in people with depressive symptoms, regardless of antidepressant usage. Future longitudinal studies including large‐scale, multi‐modal approaches are needed to further elucidate the temporal relationship between sleep disturbances and cognitive changes.

## CONFLICT OF INTEREST STATEMENT

Mrs. Young, Ms. Bernal, Dr. Zeynoun, Dr. Wiedner, Dr. Gaona, Dr. Beiser, Dr. Teixeira, Dr. Pase, and Dr. Himali have nothing to disclose. Dr. Seshadri reports consulting for Eisai and Biogen. Dr. Baril reports speaking fees from Eisai. Dr. Salardini reports speaking fees from Lilly. Author disclosures are available in the supporting information.

## CONSENT STATEMENT

All study participants signed the written informed consent form.

## Supporting information



Supporting information

Supporting information
