# Supplementary material for: Long sleep duration, cognitive performance, and the moderating role of depression: A cross‐sectional analysis in the Framingham Heart Study
Source: Alzheimers Dement. 2025 Apr 21;21(4):e70160. doi: 10.1002/alz.70160 (PMC12010301; doi:10.1002/alz.70160)
Supplement: Supplementary file 1 — Supporting information [file ALZ-21-e70160-s002.docx]

**Supplementary table 1.** Sample characteristics by age group

|  | **Whole Sample** | **≤44 years** | **≥ 45 years** |
| --- | --- | --- | --- |
| N, n (%) | 1853 | 524 (28.9) | 1329 (71.7) |
| Age at NP, years | 49.84 (9.2) | 38.77 (4.4) | 54.20 (6.5) |
| Male, n (%) | 791 (42.7) | 192 (36.6) | 599(45.1) |
| Ethnicity, n (%) |  |  |  |
| White | 1760 (95.3) | 499 (95.2) | 1261(95.2) |
| Black | 19 (1.0) | 3 (0.6) | 16 (1.2) |
| Hispanic | 33 (1.8) | 13 (2.5) | 20 (1.5) |
| Other | 35 (1.9) | 8 (1.5) | 27 (2.0) |
| Education, n (%) |  |  |  |
| Up to High School | 262 (14.1) | 51 (9.7) | 211 (15.9) |
| Some College | 532 (28.7) | 108 (20.6) | 424 (32.0) |
| College degree | 1059 (57.2) | 365 (69.7) | 694 (52.2) |
| Time interval ^a^, years | 1.69 (1.0) | 1.58 (0.9) | 1.74 (1.0) |
| APOE ɛ4 carriers, n (%) | 384 (21.8) | 118 (23.65) | 266 (21.0) |
| Total Cholesterol, mg/dL | 185.98 (35.0) | 180.4 (36.2) | 188.17 (34.3) |
| HDL, mg/dL | 60.53 (17.9) | 59.57 (16.2) | 60.9 (18.6) |
| Systolic BP, mm/Hg | 116 (14.1) | 111 (11.6) | 118 (14.5) |
| HTN treatment, n (%)  Stage 1 Hypertension, n (%) | 390 (21.1)  473 (25.7) | 35 (6.7)  48 (9.2) | 355 (26.9)  425 (32.2) |
| Triglycerides, mg/dL | 112.8 (85.8) | 108.63 (95.3) | 114.44 (81.7) |
| CRP, mg/dL | 2.83 (4.5) | 3.10 (5.5) | 2.72 (4.1) |
| Body Mass Index | 28.19 (5.8) | 27.12 (5.4) | 28.61 (5.9) |
| Depression status, n (%) | 448 (24.2) | 125 (23.9) | 323 (24.3) |
| Antidepressants usage | 315 (17.0) | 79 (15.1) | 236 (17.8) |
| Depression, CES-D ≥16 | 198 (10.7) | 62 (11.8) | 136 (10.2) |
| Control ^b^ | 1405 (75.8) | 399 (76.2) | 1006 (75.7) |
| Antidepressants usage/CES-D <16^c^ | 250 (13.5) | 63 (12.0) | 187 (14.1) |
| No Antidepressants usage/CES-D ≥ 16^d^ | 133 (7.2) | 46 (8.8) | 87 (6.6) |
| Antidepressants usage/CES-D ≥16^e^ | 65 (3.5) | 16 (3.1) | 49 (3.7) |

Abbreviations: NP= Neuropsychological testing, BP = Blood Pressure; HDL = high-density lipoprotein cholesterol; HTN = hypertension; CRP = C reactive protein. All values represent mean (SD) unless otherwise indicated by n (%).

NOTE. ^a^ time interval between self-reported sleep duration and cognitive tests. Depression groups: ^b^ Control = no antidepressants usage and no depressive symptoms (CES-D < 16); ^c^ antidepressant usage without depressive symptoms (CES-D < 16); ^d^ no antidepressant usage with depressive symptoms (CES-D ≥ 16); ^e^ antidepressant usage and with depressive symptoms (CES-D ≥ 16).

**Supplementary table 2**. Cognitive characteristics by age group

|  | **Whole Sample** | **≤44 years** | **≥ 45 years** |
| --- | --- | --- | --- |
| N, n (%) | 1853 | 524 (28.28) | 1329 (71.72) |
| Global Cognition ^a^, median [Q1, Q3] | 0.45 [-0.12,0.98] | 0.75 [0.15,1.17] | 0.34 [-0.23,0.88] |
| Trails Part A, min, median [Q1, Q3] | 0.40 [0.33,0.48] | 0.35 [0.30,0.43] | 0.42 [0.35,0.52] |
| Trails Part B, min, median [Q1, Q3] | 0.97 [0.77,1.22] | 0.85 [0.68,1.05] | 1.00 [0.80,1.28] |
| Visual Reproduction ^b^, n correct | 17.99 (4.89) | 19.75 (4.40) | 17.30 (4.90) |
| Logical Memories ^b^, n correct | 24.46 (6.74) | 25.46 (6.92) | 24.07 (6.63) |
| Similarities Test, n correct | 17.20 (3.15) | 17.28 (2.96) | 17.17 (3.22) |

Abbreviations: Min = minutes. NOTE. ^a^ weighted score units; ^b^ Sum of immediate and delayed

recall scores. All values represent mean (SD) unless otherwise indicated by median [Q1, Q3].

**Supplementary table 3.** Association between short and long sleep duration and cognitive scores after excluding participants < 45 years old (sensitivity analysis)

|  |  | **Sleep Duration Categories** | | | | |
| --- | --- | --- | --- | --- | --- | --- |
|  |  | **Average** | **Short Sleep** | | **Long Sleep** | |
|  |  | **(>6-<9 hours)** | **(≤6 hours)** | | **(≥ 9 hours)** | |
| **Cognition** | **Model** |  | **β±SE** | **p** | **β±SE** | **p** |
| Global Cognition | 1 | REF | -0.03±0.05 | 0.520 | **-0.24±0.08** | **0.004** |
|  | 2 | REF | -0.05±0.05 | 0.305 | **-0.27±0.09** | **0.002** |
| Trails Part A | 1 | REF | -0.009±0.02 | 0.622 | -0.02±0.03 | 0.469 |
|  | 2 | REF | -0.02±0.02 | 0.273 | -0.02±0.04 | 0.566 |
| Trails Part B | 1 | REF | -0.04±0.02 | 0.099 | **-0.08±0.04** | **0.039** |
|  | 2 | REF | -0.06±0.02 | 0.012 | -0.06±0.04 | 0.179 |
| Visual Reproduction | 1 | REF | -0.50±0.30 | 0.090 | **-2.05±0.52** | **<0.001** |
|  | 2 | REF | -0.53±0.30 | 0.084 | **-2.14±0.56** | **<0.001** |
| Logical Memory | 1 | REF | 0.44±0.40 | 0.279 | -1.24±0.72 | 0.087 |
|  | 2 | REF | 0.434±0.41 | 0.406 | **-2.20±0.76** | **0.004** |
| Similarities Test | 1 | REF | 0.09±0.19 | 0.661 | 0.03±0.35 | 0.925 |
|  | 2 | REF | 0.11±0.20 | 0.594 | -0.02±0.37 | 0.949 |

NOTE. Model 1 was adjusted for age at neuropsychological testing administration, age squared at neuropsychological testing administration, education, sex, time between sleep duration questionnaires and neuropsychological testing administration and cohort. Model 2 was further adjusted for hypertension, total cholesterol level to HDL ratio, triglycerides, C reactive protein level, body max index, and APOE ɛ4 status. Significant findings at p <0.05 are shown in bold.

**Supplementary table 4.** Effect Modification by depression status on the association between self-reported sleep duration and cognitive scores after excluding participants <45 years old.

|  |  | **Global Cognition** | | **Trails Part B** | | **Visual Reproduction** | | **Similarities** | |
| --- | --- | --- | --- | --- | --- | --- | --- | --- | --- |
|  | **Sleep Duration Interaction** | **p=0.009** | | **p= 0.038** | | **p=0.050** | | **p=0.006** | |
|  | | **β±SE** | **p** | **β±SE** | **p** | **β±SE** | **p** | **β±SE** | **p** |
| **Control ^a^** | **Average Sleep** | REF | | REF | | REF | | REF | |
|  | **Short Sleep** | -0.02±0.05 | 0.714 | -0.03±0.02 | 0.203 | -0.43±0.33 | 0.195 | 0.13±0.22 | 0.543 |
|  | **Long Sleep** | -0.15±0.10 | 0.157 | -0.04±0.05 | 0.481 | **-1.43±0.68** | **0.037** | 0.46±0.45 | 0.302 |
| **Antidepressants usage**  **and CES-D <16 ^b^** | Average Sleep | REF | | REF | | REF | | REF | |
|  | Short Sleep | -0.04±0.14 | 0.796 | -0.09±0.07 | 0.235 | -0.56±0.86 | 0.517 | -0.12±0.56 | 0.827 |
|  | Long Sleep | -0.15±0.16 | 0.369 | -0.07±0.08 | 0.391 | -1.15±0.96 | 0.236 | 0.04±0.63 | 0.950 |
| **No Antidepressants usage**  **and CES-D ≥ 16 ^c^** | Average Sleep | REF | | REF | | REF | | REF | |
|  | Short Sleep | -0.22±0.17 | 0.206 | -0.12±0.09 | 0.182 | -1.62±1.20 | 0.180 | -0.18±0.68 | 0.786 |
|  | Long Sleep | -0.38±0.77 | 0.623 | -0.28±0.40 | 0.484 | -5.01±5.39 | 0.356 | 0.45±3.06 | 0.884 |
| **Antidepressants usage**  **and CES-D ≥16 ^d^** | Average Sleep | REF | | REF | | REF | | REF | |
|  | Short Sleep | 0.41±0.30 | 0.177 | 0.14±0.13 | 0.260 | 0.43±1.88 | 0.821 | 2.05±1.41 | 0.156 |
|  | Long Sleep | **-1.10±0.35** | **0.004** | **-0.36±0.15** | **0.023** | **-6.59±2.24** | **0.005** | **-3.80±1.69** | **0.030** |

NOTE. Sleep duration categories: short ≤6h; average 7-8h; long ≥9h. Depression groups: ^a^ Control = no antidepressants usage and no depressive symptoms (CES-D < 16); ^b^ antidepressant usage without depressive symptoms (CES-D <16); ^c^ no antidepressant usage with depressive symptoms (CES-D ≥ 16); ^d^ antidepressant usage and with depressive symptoms (CES-D ≥ 16).

Model 1 was adjusted for age and age squared at neuropsychological testing, education, sex, time between sleep duration questionnaires and neuropsychological testing, and cohort. Significant findings at p <0.10 for interaction and at p <0.05 for the stratified model are shown in bold.

No significant interaction was found with Trails Part A (p=0.138), and Logical Memories (p=0.914).
